# RESILIENCE, COPING, AND HEALTH IN MIDDLE TO LATE LIFE

**DOI:** 10.1093/geroni/igz038.2763

**Published:** 2019-11-08

**Authors:** Roland J Thorpe, Keith E Whitfield

**Affiliations:** 1 Johns Hopkins Bloomberg School of Public Health, Baltimore, Maryland, United States; 2 Wayne State University, Detroit, Michigan, United States

## Abstract

Stress and resilience are two factors that are receiving attention as key determinants that can provide insights that underlie the deleterious effects on the overall health and well-being of individuals by influencing behavioral and biological processes. This symposium contains a collection of papers seeking to address the influence of resilience and coping on health outcomes in middle to late life adults. Tobin and Thorpe identified profiles of psychosocial resilience and examined their association with allostatic load (AL) among 283 Black men in the Nashville Stress and Health Study. Using Latent class analysis (LCA), individuals in the high resilience class had the greatest odds of high AL; high resilience worsened physical health for older but not younger Black men. Tan and colleagues explored satisfaction across life domains and correlates of satisfaction across domains in 93 Black adults. The authors report that higher satisfaction was associated with less education, less financial strain, lower depressive symptoms, and better self-rated physical health. Nguyen examined the association between everyday discrimination and generalized anxiety disorder (GAD) and whether church-based relationships buffer the negative effects of everyday discrimination on GAD among older African Americans. Using data from 670 African American respondents age 55 and older from the NSAL, the author reports, that significant interactions indicated that frequent contact with church members and high levels of subjective closeness to church members buffered against the negative effects of discrimination on GAD. These presentations collectively will bolster our knowledge of how stress and resilience impacts health disparities.

